# Survival predictors of ^177^Lu-Dotatate peptide receptor radionuclide therapy (PRRT) in patients with progressive well-differentiated neuroendocrine tumors (NETS)

**DOI:** 10.1007/s00432-021-03672-w

**Published:** 2021-06-10

**Authors:** Mina M. Swiha, Duncan E. K. Sutherland, Golmehr Sistani, Alireza Khatami, Rami M. Abazid, Amol Mujoomdar, Daniele P. Wiseman, Jonathan G. Romsa, Robert H. Reid, David T. Laidley

**Affiliations:** 1 Medical Imaging Department, Division of Nuclear Medicine, Victoria Hospital, London Health Sciences Centre, University of Western Ontario, 800 Commissioners Road East, PO Box 5010, London, ON N6A 5W9 Canada; 2 Medical Imaging Department, Division of Diagnostic Radiology, University of Western Ontario, London, ON Canada

**Keywords:** Survival predictors, Prognostic factors, Lu-Dotatate, PRRT, Neuroendocrine

## Abstract

**Purpose:**

^177^Lu-Dotatate is an emerging treatment modality for patients with unresectable or metastatic well-differentiated NETs. This study examines survival predictors in patients who received ^177^Lu-Dotatate.

**Methods:**

A retrospective single-center review was conducted, examining 47 individuals with progressive well-differentiated NETs treated with ^177^Lu-Dotatate (four induction cycles of 5.5 GBq at 10-week intervals followed by eight maintenance cycles of 3.7 GBq at 6-month intervals).

**Results:**

Median follow-up was 63.1 months with a median progression-free survival (PFS) of 34.1 months. However, median overall survival (OS) was not reached at the time of analysis. The presence of ≥ 5 bone metastases (hazard ratio HR 4.33; *p* = 0.015), non-gastroenteropancreatic (non-GEP) NETs (HR 3.22; *p* = 0.025) and development of interim ascites (HR 3.15; *p* = 0.047) independently predicted a worse OS. Patients with chromogranin A of ≥ 4 × upper limit of normal (ULN) had shorter OS (*p* < 0.001) and PFS (*p* = 0.004). Similarly, those with pre-existing ascites demonstrated a worse OS (*p* = 0.009) and PFS (*p* = 0.026). Liver metastases involving greater than 50% liver volume and the existence of unusual metastatic locations had a negative impact on OS (*p* = 0.033) and PFS (*p* = 0.026), respectively.

**Conclusion:**

High burden of skeletal and hepatic metastases, non-GEP-NETs, chromogranin A of ≥ 4 × ULN, unusual metastatic sites, pre-existing and interim ascites are predictors of poor outcomes in patients treated with ^177^Lu-Dotatate. These common indicators can be used for the risk stratification and identification of patients most likely to benefit from PRRT.

**Trial registration:**

ClinicalTrials.gov identifier: NCT02236910, Retrospectively registered on September, 2014.

## Introduction

Neuroendocrine tumors (NETs) are a heterogeneous group of neoplasms that can arise from the neuroendocrine cells in any organ but most commonly from the gastrointestinal tract and lungs (Yao et al. 2008; Oronsky et al. 2017). Embryologically, these cells originate from the neural crest and endoderm. These cells are characterized by their ability to secrete specific peptides and hormones and consequently can be syndromic (Abou Jokh et al. 2020).

There has been a gradual increase in the annual incidence of NETs by approximately sevenfold in the period between 1973 and 2012. This increase is irrespective of primary tumor site and mainly attributable to the advances in diagnostic imaging, particularly in early disease (Dasari et al. 2017). NETs typically demonstrate indolent behavior, and patients are frequently metastatic at diagnosis. Common metastatic sites include the liver, lymph nodes, peritoneum, bone, and lungs (Garcia-Carbonero et al. 2010). Uncommon sites of metastases have also been reported, including pericardium, breast tissue and soft tissue of the orbits (Srirajaskanthan et al. 2009; Naswa et al. 2013). The indolent behavior of NETs in many patients translates to a relatively longer overall survival, with a 5-year survival rate of 75% (Garcia-Carbonero et al. 2010).

The majority of NETs overexpress somatostatin receptors (SSTR). This unique characteristic of NETs allows for the development of radiopharmaceuticals with dual roles in diagnostic imaging and intervention (theranostics) (Pasieka et al. 2001). In nuclear medicine, an example of a long-standing NET radiopharmaceutical theranostic pairing is ^111^In-pentetreotide and ^123^I-metaiodobenzylguanidine for single-photon emission tomography (SPECT) functional imaging, coupled with high administered activities of ^111^In-pentetreotide and ^131^I-metaiodobenzylguanidine for therapy. Most recently, in the positron emission tomography (PET) era, diagnostic positron emitting radiopharmaceuticals, such as ^68^Ga-DOTA peptides (demonstrating even higher tumor uptake compared to SPECT radiopharmaceuticals) are combined with therapeutic ^177^Lu-DOTA peptide derivatives to forge a new line of treatment (Sadowski et al. 2016).

Surgical resection of the primary tumor and metastatic disease is the only curative treatment. However, at diagnosis, less than 10% of the patients are candidates for total resection (Modlin et al. 2010). Other interventional modalities such as liver-directed therapy including debulking surgery, radiofrequency ablation (RFA), transarterial chemoembolization (TACE) and transarterial radioembolization (TARE) should be attempted if removal of ≥ 90% of the tumor burden is feasible (Frilling et al. 2010).

The PROMID and CLARINET studies showed an improvement in progression-free survival (PFS) in patients with advanced gastroenteropancreatic neuroendocrine tumors (GEP-NETs) using octreotide long-acting repeatable (LAR) and lanreotide, respectively (Rinke et al. 2009; Caplin et al. 2016). A few additional systemic therapeutic options are available for progressive NETs including targeted therapies such as everolimus and sunitinib, capecitabine/temozolomide and other chemotherapeutic agents. However, most show limited efficacy and each has its own set of side effects (Vaughan et al. 2018).

Peptide receptor radionuclide therapy (PRRT) is a novel treatment for advanced NETs which exploits somatostatin analogues (SSA) in combination with β-emitters such as ^90^Y and ^177^Lu, to target cells with SSTR overexpression (Kwekkeboom et al. 2005). Recently, the phase III randomized trial NETTER-1 demonstrated that patients with low and intermediate grade advanced midgut NETs who received ^177^Lu-DOTA^0^–Tyr^3^–octreotate (^177^Lu-Dotatate) had significant PFS improvement in comparison to patients who received high dose of octreotide LAR (Strosberg et al. 2017). Accordingly, ^177^Lu-Dotatate, a PRRT agent, was approved for patients with progressive well-differentiated midgut NETs in the United States of America and Canada (Millburn 2018). The response predictors of this novel treatment have not yet been well described in the literature. Moreover, while ^177^Lu-Dotatate is reported to be cost-effective at a national level (Smith-Palmer et al. 2021), the unit cost is sufficiently high to warrant further investigation to identify those most likely to benefit from treatment. In this report, we describe several prognostic factors of adverse outcome and recurrent disease. In comparison to the NETTER-1 trial which used a shorter course of high activity of ^177^Lu-Dotatate, this phase II trial aimed primarily to assess the efficacy and safety of an extended course of lower ^177^Lu-Dotatate activity, and to assess the efficacy of ^177^Lu-Dotatate in a broad spectrum of primary tumors.

## Materials and methods

### Patients

We retrospectively analyzed a cohort of 47 patients who received ^177^Lu-Dotatate in an ongoing phase II, open-label, single-arm registry study. All patients with NETs of variable primary sites, naive to ^177^Lu-Dotatate PRRT, were enrolled in the period between July 2014 and October 2016. All patients were reviewed and deemed suitable for PRRT by a dedicated NET Tumor Board. The inclusion criteria were progressive disease, Ki-67 of ≤ 20%, documented tumor positivity for SSTR on SPECT functional imaging (Krenning score of ≥ 3) within 4 months of enrollment, life expectancy of > 6 months, hemoglobin of ≥ 90 g/dL, thrombocytes of ≥ 100 × 10^9^/L, total leukocyte count of ≥ 3 × 10^9^/L, glomerular filtration rate of > 50 mL/min/1.73 m^2^, bilirubin of ≤ 3 × upper limit of normal (ULN) and Eastern Cooperative Oncology Group (ECOG) performance score 0–2 measured within 1 month of enrollment. The study excluded patients with potentially resectable tumors, non-irradiated brain metastases, poor bone marrow reserve secondary to prior extensive bone irradiation, co-existing malignancies and uncontrolled diabetes mellitus (fasting blood glucose ≥ 3 × ULN within 12 weeks of enrollment). Pregnant and nursing females were also excluded from the study.

### Treatment protocol

Thirty minutes after premedication with antiemetics, an amino acid solution (1 L of 2.5% arginine and 2.5% lysine) was administered through a slow intravenous infusion over 4 h. Thirty minutes following the initiation of the amino acid infusion, ^177^Lu-Dotatate was co-administered over 30–45 min. The induction phase included four cycles of 5.5 GBq (150 mCi) ^177^Lu-Dotatate administered approximately over 10-week intervals (8–12 weeks). Disease control after the induction phase was defined as the sum total of patients with complete response, partial response and stable disease by Response Evaluation Criteria in Solid Tumors (RECIST 1.1) (Therasse et al. 2000). Those patients who had achieved disease control were then started on the maintenance phase of therapy which included eight cycles of 3.7 GBq (100 mCi) ^177^Lu-Dotatate over approximately 6-month intervals for up to 4 years or until disease progression, depending on whichever occurred earlier.

Most patients 46/47 (98%) continued to receive supportive care with SSA, with the exception of one patient with a pheochromocytoma. Long-acting SSA were withheld for at least 1 week prior to therapy and resumed one 1 week after the treatment, while short acting SSA were withheld for 24 h before and resumed 24 h after treatment.

Both functional and anatomical imaging were used for the assessment of treatment response. Post-therapy whole body planar scintigraphy with SPECT/CT was performed at 4 and 24 h. A dedicated CT was performed 3–4 months after the completion of the induction phase and 4 months after each maintenance cycle. The CT images were reviewed by two expert NET radiologists, while the functional imaging scans were assessed by an expert NET nuclear medicine physician.

### Definitions of survival outcomes and covariables

Overall survival (OS) was defined as the time from date of enrollment to death due to any cause. PFS was defined as the time from date of enrollment to disease progression according to RECIST 1.1 criteria. Tumor functionality was determined by elevated 24-h urine 5-hydroxy indoleacetic acid or the development of typical carcinoid syndrome symptoms such as intermittent flushing, diarrhea and/or bronchospasm. Baseline chromogranin A and lactate dehydrogenase (LDH) levels were obtained within 4 weeks prior to PRRT initiation. Liver metastatic volume and the number of bone metastases were assessed on both anatomical and functional imaging. Pre-existing ascites refers to the presence of peritoneal fluid on baseline imaging prior to the start of ^177^Lu-Dotatate, while interim ascites was defined as newly accumulating peritoneal fluid either during the treatment period with ^177^Lu-Dotatate or after its cessation. Typical sites of metastases were defined as liver, lung, bone, peritoneum and lymph nodes. All other metastatic sites were considered unusual/uncommon.

### Statistical analysis

All statistical analyses were performed using IBM SPSS (macOS 10.15). Continuous variables were described as mean ± standard deviation while categorical data as percentage. The OS and PFS were assessed using Kaplan–Meier analysis and Log-rank test was used to compare survival curves between the different groups. Cox regression analysis was used for both univariate and multivariate analyses of the different covariables with the outcomes. Only covariates with a *p* value of < 0.05 in the univariate model were entered into the multivariate model to evaluate for independent variables. A *p* value of < 0.05 was considered significant.

## Results

### Baseline clinical characteristics

In total, 47 patients were included in the study with a mean age of 62 ± 10 years (range 34–83 years) at enrollment. Among those enrolled, 28/47 (59.6%) were men. Other baseline characteristics are shown in Table 1.

**Table 1:** Patient characteristics

Variable	Number (%)
Gender	
Male	28 (59.6)
Female	19 (40.4)
Primary tumor	
Midgut	20 (42.6)
Pancreas	13 (27.7)
Duodenum/foregut	1 (2.1)
Rectum/hindgut	2 (4.3)
Lung	3 (6.4)
Ovary	1 (2.1)
Pheochromocytoma	1 (2.1)
Kidney	1 (2.1)
Eustachian tube	1 (2.1)
Thymus gland	1 (2.1)
Unknown primary	3 (6.4)
Tumor grade	
Grade 1	15 (31.9)
Grade 2	32 (68.1)
Ki-67	
1–10%	35 (74.5)
11–20%	12 (25.5)
Baseline chromogranin A	
Normal (≤ 110 ng/mL)	13 (27.7)
Elevated (> 110 ng/mL)	34 (72.3)
Functionality	
Non-functioning	31 (66)
Functioning	16 (34)
Baseline LDH	
Normal (≤ 216 U/L)	39 (83)
Elevated (> 216 U/L)	8 (17)
Liver metastases (% liver volume)	
No	9 (19.1)
< 25%	13 (27.7)
25–50%	14 (29.8)
> 50%	11 (23.4)
Bone metastases (number of bone deposits)	
No	24 (51.1)
1–4	12 (25.5)
5–9	4 (8.5)
≥ 10	7 (14.9)
Lung metastases	
No	41 (87.2)
Yes	6 (12.8)
Abdominal lymphadenopathy	
No	16 (34)
Yes	31 (66)
Peritoneal metastases	
No	41(87.2)
Yes	6 (12.8)
Pre-existing ascites	
No	43 (91.5)
Yes	4 (8.5)
Interim ascites	
No	31 (66)
Yes	16 (34)
Pleural effusion	
No	44 (93.6)
Yes	3 (6.4)
Unusual metastatic sites	
No	41 (87.3)
Pericardial	2 (4.3)
Brain	1 (2.1)
Orbit	1 (2.1)
Thyroid gland	1 (2.1)
Breast	1 (2.1)
Primary tumor resection	
No	12 (25.5)
Yes	35 (74.5)
Long-acting SSA	
No	1 (2.1)
Standard dose octreotide LAR (30 mg every 4 weeks)	29 (61.7)
Escalated dose octreotide LAR (60 mg every 4 weeks)	11 (23.4)
Lanreotide (120 mg every 4 weeks)	6 (12.8)
Chemotherapy/targeted therapy	
No	34 (72.3)
Platinum-based chemotherapy	8 (17)
Capecitabine–Temozolomide	2 (4.3)
Everolimus	2 (4.3)
Sunitinib	1 (2.1)
Liver-directed therapy	
No	30 (63.8)
Hepatectomy ± RFA or TACE	9 (19.1)
TACE	5 (10.7)
RFA	3 (6.4)
External beam radiation	
No	42 (89.4)
Yes	5 (10.6)

*LDH* lactate dehydrogenase, *SSA* somatostatin analogues, *LAR* long-acting repeatable, *RFA* radiofrequency ablation, *TACE* transarterial chemoembolization

Midgut NETs were the most common primary tumor, being present in 20/47 (42.6%), followed by pancreatic NETs in 13/47 (27.7%) and non-GEP-NETs in 11/47 (23.4%). Nearly all patients 46/47 (98%) completed the induction phase of ^177^Lu-Dotatate, with the exception of a single patient with bronchial NET who developed disease progression after the second cycle. Another patient from outside the province was lost to follow-up after induction therapy completion and was excluded from further analysis.

After the induction phase, the majority of the patients 40/47 (85.1%) achieved disease control with partial response in 15/47 (31.9%) and disease stability in 25/47 (53.1%) by RECIST 1.1 criteria. However, 7/47 (14.9%) patients had disease progression. The treatment was generally well tolerated; no grade 3 or 4 toxicity was reported according to the National Cancer Institute Common Terminology Criteria for Adverse Events, version 5.0.

The median follow-up period was 63.1 months (95% confidence interval CI 59.4–66.8) calculated by reverse Kaplan–Meier analysis. At the time of analysis, 37/46 (80.4%) patients had developed disease progression including progression, in either induction and/or maintenance phases, whereas 9/46 (19.6%) patients continued to have disease control on maintenance therapy. The median cumulative activity including induction phase was 25.9 GBq with a median of six cycles administered. 20/46 (43.5%) patients were deceased at the time of analysis and all showed evidence of disease progression.

### Overall survival analysis

**Fig. 1:**
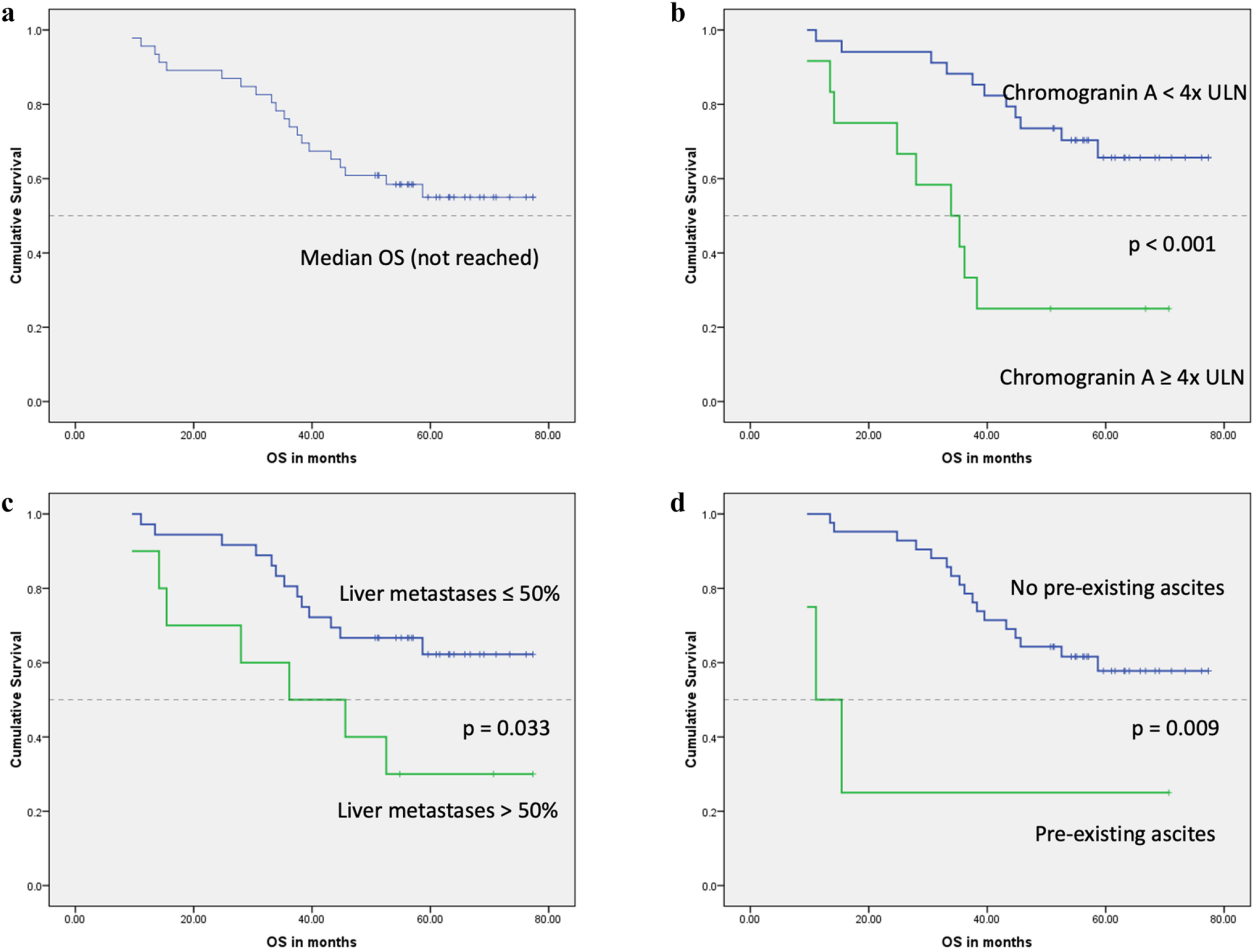
Kaplan–Meier curves for OS: **a** the median OS for the study group had not been reached. **b** OS by chromogranin A; there was a significant difference in the OS between patients with chromogranin A of ≥ 4 × ULN and those with chromogranin A of < 4 × ULN (*p* < 0.001). **c** OS survival by burden of liver metastases; there was a significant difference in the OS between patients with liver metastases of > 50% liver volume compared to those with ≤ 50% liver volume (*p* = 0.033). **d** OS by pre-existing ascites; patients with pre-existing ascites prior PRRT initiation had a shorter OS compared to those without pre-existing ascites (*p* = 0.009)

**Fig. 2:**
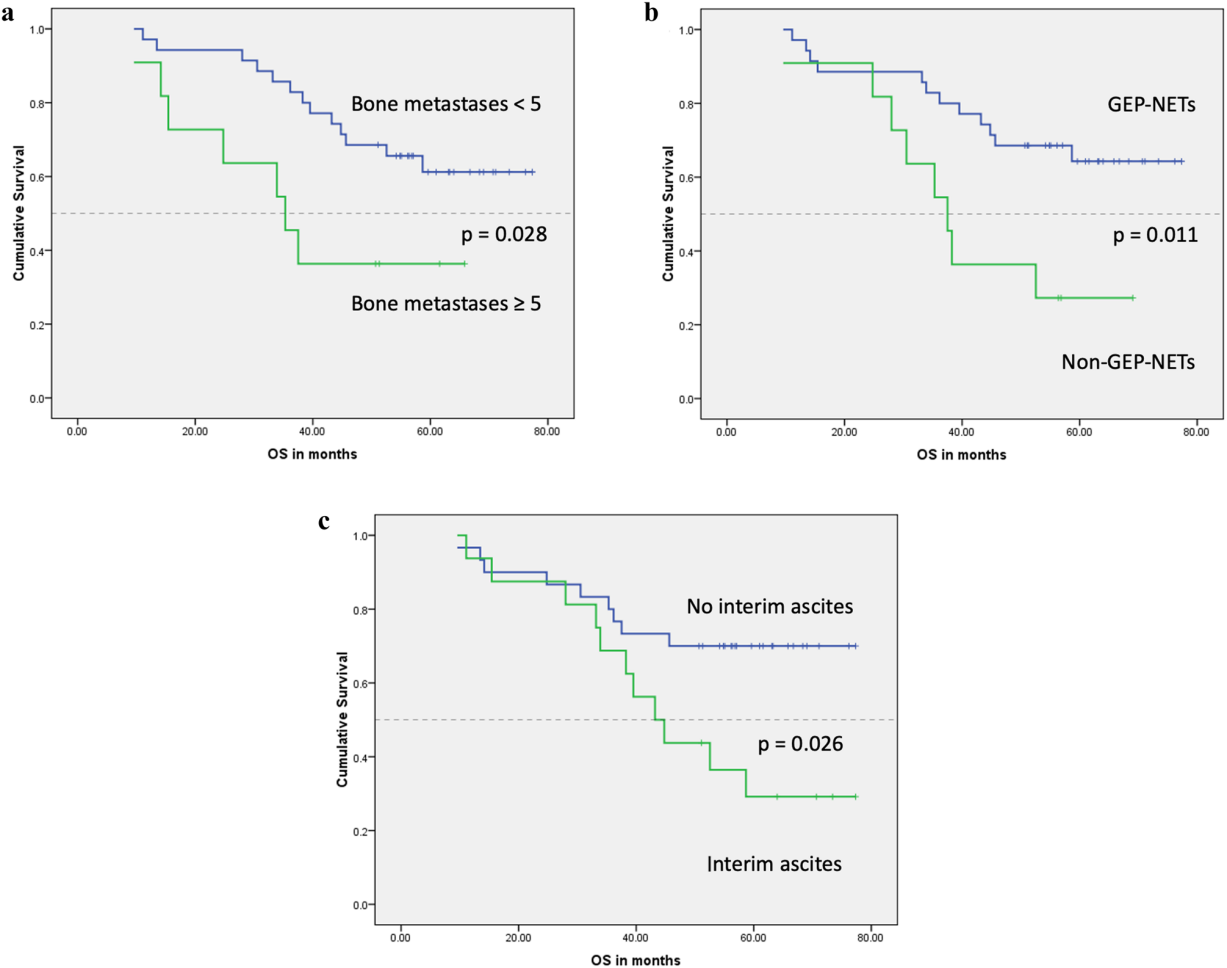
Kaplan–Meier curves for OS: **a** OS by number of bone metastases; there was a significantly shorter OS in patients with (≥ 5) bone deposits compared to patients with (0–4) bone deposits (*p* = 0.028). **b** OS by primary tumor site; a longer OS was observed in patients with GEP-NETs vs non-GEP-NETs (*p* = 0.011). **c** OS by interim ascites; patients who developed ascites during the period of treatment with ^177^Lu-Dotatate or after its cessation had a shorter OS (*p* = 0.026)

At the time of the analysis, the median OS for the study group had not been reached, while the mean OS was 57.4 months (95% CI 50.5–64.4). However, in the subgroup analysis, we found that patients with high metastatic burden within the liver of greater than 50% liver volume and those with five or more bone deposits have poor median OS (36.2 months vs. not reached; *p* = 0.033) and (35.3 months vs. not reached; *p* = 0.028), respectively. In addition, we found that OS was inversely related to elevated chromogranin A of ≥ 4 × ULN (33.9 months vs. not reached; *p* < 0.001), pre-existing ascites (11.1 months vs. not reached; *p* = 0.009), development of interim ascites (43.2 months vs. not reached; *p* = 0.026) and non-GEP-NETs (37.5 months vs. not reached; *p* = 0.011) (Table 2; Figs. 1 and 2).

**Table 2:** Kaplan–Meier analysis of OS

Variable	Categories	No. of patients	No of patients deceased	Median OS (months)	95% CI	*p* value
Chromogranin A*	< 4 × ULN	34	11	Not reached		< 0.001
	≥ 4 × ULN	12	9	33.9	21.5–46.3	
Pre-existing ascites*	No	42	17	Not reached		0.009
	Yes	4	3	11.1	5.3–16.8	
Interim ascites*	No	30	9	Not reached		0.026
	Yes	16	11	43.2	32.9–53.5	
Liver metastases*	≤ 50%	36	13	Not reached		0.033
	> 50%	10	7	36.2	8.8–63.5	
Bone metastases*	< 5 sites	35	13	Not reached		0.028
	≥ 5 sites	11	7	35.3	21.6–49.1	
Primary tumor site*	GEP-NETs	35	12	Not reached		0.011
	Non-GEP-NETs	11	8	37.5	29.2–45.9	
Unusual metastatic sites	No	40	16	Not reached		0.12
	Yes	6	4	35.3	23.9–46.7	
Baseline LDH	Normal	37	15	Not reached		0.38
	Elevated	8	5	45.6	24.8–66.5	
Gender	Male	28	13	Not reached		0.55
	Female	18	7	Not reached		
Tumor grade	Grade 1	15	8	Not reached		0.53
	Grade 2	31	12	58.7		
Ki-67	1–10%	33	13	Not reached		0.28
	11–20%	12	7	45.6	14–77.3	
Functionality	Non-functioning	30	12	Not reached		0.60
	Functioning	16	8	58.7		
Lung metastases	No	40	16	Not reached		0.30
	Yes	6	4	43.2	15.2–71.3	
Abdominal lymphadenopathy	No	15	6	Not reached		0.70
	Yes	31	14	Not reached		
Peritoneal metastases	No	40	15	Not reached		0.06
	Yes	6	5	39.5	33.6–45.4	
Pleural effusion	No	43	18	Not reached		0.24
	Yes	3	2	35.3	0–74.1	
Primary tumor resection	No	12	5	Not reached		0.70
	Yes	34	15	Not reached		
SSA	No	1	1	24.8		0.12
	Standard dose LAR	28	12	Not reached		
	Escalated dose LAR	11	4	Not reached		
	Lanreotide	6	3	38.3		
Chemotherapy/targeted therapy	No	34	13	Not reached		0.23
	Yes	12	7	45.6	23.6–67.7	
Liver-directed therapy	No	30	14	58.7		0.64
	Yes	16	6	Not reached		

*OS* overall survival, *CI* confidence interval, *ULN* upper limit of normal, *GEP-NETs* gastroenteropancreatic neuroendocrine tumors, *LDH* lactate dehydrogenase, *SSA* somatostatin analogues, *LAR* long-acting repeatable

*Denotes that there was significant difference between the two subgroups (*p* < 0.05)

Univariate regression analysis also demonstrated high metastatic liver burden of > 50% liver volume (hazard ratio HR 2.6, 95% CI 1.05–6.61; *p* = 0.04), five or greater skeletal metastatic deposits (HR 2.72, 95% CI 1.07–6.88; *p* = 0.035), high level of baseline chromogranin A of ≥ 4 × ULN (HR 4.34, 95% CI 1.76–10.7; *p* = 0.001), pre-existing ascites (HR 4.51, 95% CI 1.3–15.59; *p* = 0.017), development of interim ascites (HR 2.64, 95% CI 1.09–6.38; *p* = 0.032) and non-GEP-NETs (HR 3.07, 95% CI 1.24–7.6; *p* = 0.016) to be risk factors of poor OS (Table 3).

**Table 3:** Univariate and multivariate Cox regression analysis for OS

Variable	Univariate Cox regression for OS				Multivariate Cox regression for OS			
		95% CI for HR		*p* value		95% CI for HR		*p* value
	HR	Lower	Upper		HR	Lower	Upper	
Chromogranin A ≥ 4 × ULN	4.342	1.763	10.696	0.001	2.117	0.761	5.892	0.151
Pre-existing ascites	4.509	1.304	15.591	0.017	1.581	0.275	9.073	0.608
Interim ascites*	2.636	1.089	6.380	0.032	3.145	1.013	9.765	0.047
Liver metastases > 50%	2.630	1.046	6.611	0.040	1.633	0.452	5.907	0.454
≥ 5 bone deposits*	2.717	1.073	6.884	0.035	4.330	1.332	14.075	0.015
Non-GEP-NETs*	3.067	1.237	7.603	0.016	3.216	1.157	8.945	0.025

*OS* overall survival, *HR* hazard ratio, *CI* confidence interval, *ULN *upper limit of normal, *GEP-NET* gastroenteropancreatic neuroendocrine tumors

*Denotes that these prognostic factors were significant in multivariate regression analysis as independent factors

Importantly, multivariate regression analysis indicated that having five or more bone metastases (HR 4.33, 95% CI 1.33–14.08, *p* = 0.015), non-GEP-NETs (HR 3.22, 95% CI 1.16–8.95; *p* = 0.025) and the development of interim ascites (HR 3.15, 95% CI 1.01–9.77, *p* = 0.047) are independent predictors of poor OS.

### Progression-free survival analysis

**Fig. 3:**
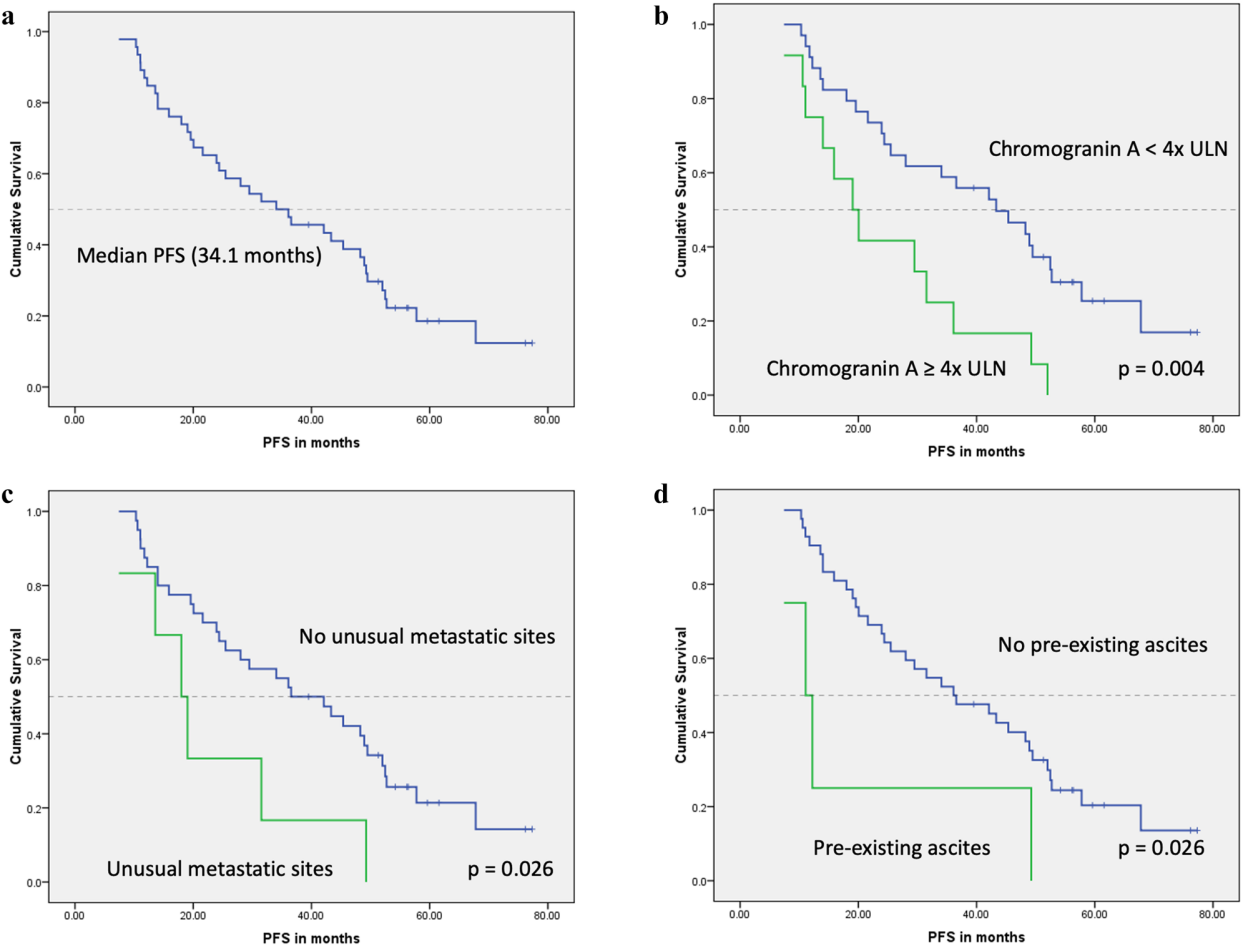
Kaplan–Meier curves for PFS: **a** the median PFS of the study group was 34.1 months; **b** PFS by Chromogranin A; there was a significant difference in the PFS between patients with chromogranin A of ≥ 4 × ULN compared to those with chromogranin A of < 4 × ULN (*p* = 0.004); **c** PFS by presence of uncommon sites of metastases; patients with uncommon/unusual metastatic sites had a poor PFS (*p* = 0.026); **d** PFS by pre-existing ascites; patients with pre-existing ascites prior PRRT initiation had a shorter PFS compared to those without pre-existing ascites (*p* = 0.026)

Kaplan–Meier analysis showed that for the study group, the median PFS was 34.1 months (95% CI 18.6–49.5). PFS was significantly shorter in patients with chromogranin A of ≥ 4 × ULN (19 vs. 43.3 months; *p* = 0.004), pre-existing ascites (11.1 vs. 36.1 months; *p* = 0.026) and presence of unusual sites of metastases (18 vs. 36.6 months; *p* = 0.026) (Table 4; Fig. 3).

**Table 4:** Kaplan–Meier analysis of PFS

Variable	Categories	No. of patients	No of patients progressed	Median PFS (months)	95% CI	*p* value
Chromogranin A*	< 4 × ULN	34	25	43.3	27.4–59.3	0.004
	≥ 4 × ULN	12	12	19	12–26.1	
Pre-existing ascites*	No	42	33	36.1	21.7–50.5	0.026
	Yes	4	4	11.1	6.4–15.8	
Unusual metastatic sites*	No	40	31	36.6	22.6–50.6	0.026
	Yes	6	6	18	11.4–24.5	
Primary tumor site	GEP-NETs	35	27	42.1	26.1–58.0	0.14
	Non-GEP-NETs	11	10	23.9	12.7–35.2	
Baseline LDH	Normal	37	31	34.1	23.9–44.3	0.51
	Elevated	8	6	24.4	0–59.5	
Gender	Male	28	25	28	9.2–46.8	0.10
	Female	18	12	49.3	15.3–83.2	
Tumor grade	Grade 1	15	12	42.1	17.6–66.6	0.74
	Grade 2	31	25	34.1	20.8–47.3	
Ki-67	1–10%	33	26	36.6	23.6–49.5	0.67
	11–20%	12	10	23.9	19.2–28.7	
Functionality	Non-functioning	30	24	28	12.3–43.7	0.93
	Functioning	16	13	36.6	17.3–55.9	
Liver metastases	≤ 50%	36	27	36.6	19.7–53.4	0.09
	> 50%	10	10	20.1	11.8–28.3	
Bone metastases	< 5 sites	35	28	43.3	27.5–59.2	0.09
	≥ 5 sites	11	9	15.9	11.1–20.6	
Lung metastases	No	40	31	36.1	16.9–55.3	0.16
	Yes	6	6	28	4.3–51.8	
Abdominal lymphadenopathy	No	15	14	25.5	12.7–38.3	0.25
	Yes	31	23	36.6	10.5–62.6	
Peritoneal metastases	No	40	32	36.1	19.8–52.4	0.35
	Yes	6	5	28	9.4–46.6	
Pleural effusion	No	43	34	36.1	18.2–54	0.39
	Yes	3	3	31.5	0–64.3	
Primary tumor resection	No	12	9	36.6	17.4–55.8	0.51
	Yes	34	28	25.5	8.1–42.9	
SSA	No	1	1	15.9		0.43
	Standard dose LAR	28	22	36.6	14.9–58.2	
	Escalated dose LAR	11	10	36.1	16.5–55.7	
	Lanreotide	6	4	19.6	5.7–33.4	
Chemotherapy/targeted therapy	No	34	25	34.1	10.7–57.4	0.19
	Yes	12	12	31.5	19.5–43.5	
Liver-directed therapy	No	30	23	34.1	15.8–52.3	0.71
	Yes	16	14	25.5	0–53.9	

*PFS* progression-free survival, *CI* confidence interval, *ULN* upper limit of normal, *GEP-NET* gastroenteropancreatic neuroendocrine tumors, *LDH* lactate dehydrogenase, *SSA* somatostatin analogues, *LAR* long-acting repeatable

*Denotes that there was significant difference between the two subgroups (*p* < 0.05)

Univariate regression analysis confirmed that baseline chromogranin A of ≥ 4 × ULN (HR 2.78, 95% CI 1.35–5.72; *p* = 0.005), presence of ascites (HR 3.13, 95% CI 1.09–9.03; *p* = 0.034) and presence of unusual sites of metastases (HR 2.67, 95% CI 1.09–6.58; *p* = 0.033) are significant predictors for poor PFS. Furthermore, multivariate regression analysis showed that chromogranin A of ≥ 4 × ULN tends toward significance as an independent risk factor for shorter PFS (HR 2.35, 95% CI 0.99–5.57; *p* = 0.054) (Table 5).

**Table 5:** Univariate and multivariate Cox regression analysis of PFS

Variable	Univariate Cox regression for PFS				Multivariate Cox regression for PFS			
		95% CI for HR		*p* value		95% CI for HR		*p* value
	HR	Lower	Upper		HR	Lower	Upper	
Chromogranin A ≥ 4 × ULN	2.781	1.351	5.724	0.005	2.345	0.987	5.574	0.054
Pre-existing ascites	3.133	1.087	9.026	0.034	1.580	0.345	7.234	0.556
Unusual metastatic sites	2.671	1.085	6.576	0.033	1.150	0.288	4.590	0.843

*PFS* progression-free survival, *CI* confidence interval, *HR* hazard ratio, *ULN* upper limit of normal

## Discussion

In this study, we analyzed the survival predictors associated with ^177^Lu-Dotatate therapy in a cohort of 47 patients with well-differentiated NETs from different primary sites. We found that OS was inversely related to non-GEP-NETs, chromogranin A of ≥ 4 × ULN, pre-existing ascites, development of interim ascites, high tumor burden within the liver of > 50% liver volume and five or more bone metastases. High burden of skeletal metastases (five or more), non-GEP-NETs and interim ascites were independent predictors for shorter OS. Furthermore, Chromogranin A of ≥ 4 × ULN, pre-existing ascites and presence of unusual sites of metastases were significantly associated with shorter PFS.

NETTER-1 determined the cumulative PFS at 20 months to be 65.2% in a cohort of 110 patients with midgut well-differentiated NETs after treatment with 4 cycles of 7.4 GBq (200 mCi) ^177^Lu-Dotatate (Strosberg et al. 2017). Ezziddin et al. (2014) also calculated the median PFS of 74 patients with grade 1 and 2 GEP-NETs who received four doses of 7.9 GBq ^177^Lu-Dotatate at 26 months. Our study showed a higher cumulative PFS at 20 months of 67.4% and longer median PFS at 34.1 months. The exact reasons for this survival benefit in our study are not entirely clear. However, it might relate to the longer course of ^177^Lu-Dotatate, which could have increased the tumor radiosensitivity to β-particles by allowing for reoxygenation of the hypoxic tumor cells and redistribution of the tumor cells into more radiosensitive phases of the cell cycle (Bodei et al. 2020; Sistani et al. 2020).

The impact of bone metastases on the prognosis of NETs has been addressed in a retrospective analysis of 314 patients with well-differentiated NETs. The investigators found that patients with bone metastases (11%) had a shorter median OS of 52 months compared to 98 months for patients with metastatic NETs without skeletal involvement, regardless of the treatment modality (Kavecansky et al. 2015). It should be noted, however, that ^177^Lu-Dotatate was not used in this study. Our results are similar to those reported by Abou Jokh et al. (2020) who demonstrated that the presence of bone metastases was associated with a shorter OS in patients with well-differentiated NETs who received ^177^Lu-Dotatate. Notably, the prevalence of skeletal metastases was greater in our study population (48.9%) compared to that reported by Abou Jokh (27.8%). To the best of our knowledge, our current study is the first study to identify a numerical cut point of five or more bone deposits as an independent predictor of shorter OS (35.3 months) in patients with well-differentiated NETs who received ^177^Lu-Dotatate.

Previous epidemiological studies have analyzed the prognostic impact of the site of the primary tumor (Dasari et al. 2017; Garcia-Carbonero et al. 2010). Data addressing its importance in patients treated with PRRT is limited since only GEP-NETs have been included in the most recent reports. Approximately one-quarter (23%) of our patients had non-GEP-NETs, with poor survival outcomes compared to those with GEP-NETs. Larger studies are needed to evaluate the efficacy of ^177^Lu-Dotatate in non-GEP-NETs compared to GEP-NETs.

Ascites in patients with NETs can be due to peritoneal carcinomatosis, advanced hepatic metastases with secondary portal hypertension, congestive heart failure secondary to carcinoid heart disease and lymphatic invasion by tumor (Warner et al. 2011). In this study, 8.5% of the patients had mild ascites detected on baseline scans. Although this did not affect their performance status (ECOG of ≤ 2), it was associated with a significantly poorer PFS and OS compared to the other patients. Likewise, 34% of patients developed interim ascites which was also associated with worse outcomes. Again, this is the first study highlighting the prognostic impact of ascites in patients with NETs. Consequently, ascites might justifiably be considered as an early sign of disease progression and treatment resistance.

Chromogranin A is a glycoprotein synthesized and stored in the neuroendocrine cells and can be used as a tumor marker in NETs reflecting tumor burden (Pasieka et al. 2001). The negative prognostic impact of elevated chromogranin A in NETs has been studied relative to different primary sites, treatment modalities and cutoff values. The phase II trial RADIANT-1 showed that elevated baseline chromogranin A in patients with advanced pancreatic NETs who received everolimus was associated with poor outcome (Yao et al. 2011). Similarly, Bergestuen et al. (2009) concluded that baseline chromogranin A of ≥ 6.2 × ULN in patients with small bowel NETs was associated with a shorter OS of 6.3 years compared to 16.4 years regardless of the treatment modality. However, none of the previous studies examined a cutoff value of chromogranin A in patients treated with ^177^Lu-Dotatate. In our study, elevated chromogranin A of ≥ 4 × ULN was associated with significantly shorter PFS and OS.

Studies have shown that up to three-quarters of the patients with NETs develop hepatic metastases, which have a poor impact on prognosis regardless of the primary tumor site (Frilling et al. 2010). The presence of a liver metastatic burden of > 50% liver volume was considered a predictor of shorter OS (Laskaratos et al. 2018), and this was also noted in the present study. The present study is the first to analyze the impact of uncommon metastatic sites on prognosis. Unusual metastatic sites were found in 12.8% and were associated with shorter PFS. We observed that the existence of uncommon metastatic sites was most commonly associated with extensive metastatic disease in liver, bone and/or lungs.

Lactate dehydrogenase enzyme (LDH) is essential for the reduction of pyruvate into lactate in tumor cells and thus, LDH levels are directly proportional to the glucose consumption by tumor cells (Petrelli et al. 2015). Elevated LDH has previously been associated with poor prognosis in the sitting of poorly differentiated NETs (Sorbye et al. 2013; Galvano et al. 2020). In this study, LDH level was not found to significantly affect survival in patients with well-differentiated NETs.

### Study limitations

This study is a single-center study with a small number of patients. The functional imaging was performed mainly with ^111^In-pentetreotide, a well established but relatively less sensitive radiotracer in comparison to ^68^Ga-Dotatate. The latter was unavailable at our center. The new category of well-differentiated grade 3 pancreatic NETs (Ki-67 > 20%) was not included in our analysis; thus, our results cannot be generalized to this group.

## Conclusion

In summary, we demonstrate that in patients with well-differentiated NETs treated with PRRT, the existence of five or more bone metastases, non-GEP-NETs and the development of interim ascites are independent prognostic factors for shorter OS. Elevated chromogranin A of ≥ 4 × ULN and the presence of pre-existing ascites have a negative impact on both PFS and OS. Moreover, the presence of uncommon sites of metastases and high burden of liver metastases of greater than 50% liver volume are associated with poor PFS and OS, respectively. This adds to our knowledge about ^177^Lu-Dotatate therapy in various NET groups and may help practitioners when assessing who might stand to benefit the most from this therapy.

## Data Availability

The datasets generated and analyzed during the current study are available from the corresponding author on reasonable request. Code availability IBM SPSS software (version 10.15 for macOS).

## Abbreviations

CI: Confidence interval
GEP-NETs: Gastroenteropancreatic neuroendocrine tumors
HR: Hazard ratio
LAR: Long-acting repeatable
NETs: Neuroendocrine tumors
OS: Overall survival
PFS: Progression-free survival
PRRT: Peptide receptor radionuclide therapy
SPECT: Single-photon emission tomography
SSA: Somatostatin analogues
SSTR: Somatostatin receptors
ULN: Upper limit of normal
